# Determinants and Health Outcomes of Digital Health Literacy in Patients With Cardiovascular Disease: Systematic Review and Meta-Analysis

**DOI:** 10.2196/89102

**Published:** 2026-03-24

**Authors:** Eun-Jung Kim, Da-Young Kim, Ha-Jin Kim, Youn-Jung Son

**Affiliations:** 1 Graduate School of Nursing Chung-Ang University Seoul Republic of Korea; 2 Department of Nursing Asan Medical Center Seoul Republic of Korea; 3 Red Cross College of Nursing Chung-Ang University Seoul Republic of Korea

**Keywords:** cardiovascular disease, digital health, health literacy, systematic review, meta-analysis

## Abstract

**Background:**

With expansion of technology-enabled care, digital health literacy (DHL) has become integral to effective cardiovascular disease (CVD) management. However, quantitative evidence regarding determinants and health outcomes of DHL in CVD remains limited and heterogeneous, necessitating comprehensive evidence synthesis.

**Objective:**

This study aimed to (1) estimate DHL levels, (2) synthesize DHL-associated factors, and (3) examine DHL-related health outcomes in CVD.

**Methods:**

A systematic review and meta-analysis of DHL in adults with CVD was conducted per PRISMA (Preferred Reporting Items for Systematic Reviews and Meta-Analyses) 2020 guidelines. PubMed, Embase, Cochrane CENTRAL, CINAHL, Scopus, Web of Science, and Google Scholar were searched for peer-reviewed studies published between 2006 and January 31, 2026. Quantitative studies enrolling adults with CVD, which reported a measure of DHL were included. Studies focusing exclusively on primary cerebrovascular disease and non–peer-reviewed articles were excluded. Risk of bias (ROB) was assessed using the Appraisal Tool for Cross-Sectional Studies tool, the Newcastle-Ottawa Scale, the Revised Cochrane Risk-of-Bias Tool for Randomized Trials, and the Risk of Bias in Nonrandomized Studies of Interventions. Certainty of evidence was evaluated using the Grading of Recommendations Assessment, Development, and Evaluation approach. Pooled mean eHealth Literacy Scale (eHEALS) scores were synthesized using a random-effects meta-analysis. Heterogeneity was quantified using the *I*^2^ statistic and 95% prediction intervals.

**Results:**

Twenty studies involving 8581 adults with CVD were included. The overall pooled mean eHEALS score was 24.26 (95% CI 21.19-27.32), with substantial heterogeneity (*I*^2^=98.4%; *τ*^2^=15.55; *τ*=3.94) and a wide 95% prediction interval (14.66-33.85). Lower DHL was consistently associated with older age, lower educational attainment, female sex, limited social support, and less experience with digital technologies. Higher DHL was associated with more favorable health-related outcomes, including health behaviors, better quality of life, and greater use and acceptance of digital health technologies. Subgroup analyses showed no statistically significant differences in DHL by region, disease type, or age group. The certainty of evidence was rated as low to very low, and substantial heterogeneity persisted across analyses.

**Conclusions:**

Our findings underscore DHL as a foundational capability for digitally supported self-management in CVD care and reveal disparities associated with age and socioeconomic factors. By integrating evidence on DHL levels, associated factors, and DHL-related health outcomes in CVD populations, this review provides a more comprehensive, clinically relevant understanding of DHL beyond studies relying on a single instrument (eg, eHEALS) or examining isolated domains. DHL appears to be a context-dependent competency shaped by broader structural and social determinants. From a clinical and health system perspective, digital health interventions should be accompanied by structured digital inclusion strategies, including routine assessment of DHL and care delivery to patients’ digital capacities. Further longitudinal and interventional studies are warranted to clarify the causal pathways linking DHL to health outcomes in adults with CVD and to incorporate provider- and system-level perspectives beyond individual-level assessments.

**Trial Registration:**

PROSPERO International Prospective Register of Systematic Reviews CRD420251068000; https://www.crd.york.ac.uk/PROSPERO/view/CRD420251068000

## Introduction

Cardiovascular disease (CVD) encompasses disorders of the heart and vasculature, including angina, myocardial infarction, stroke, heart failure, arrhythmia, and valvular heart disease [1]. CVD is typically chronic and requires lifelong management. Effective patient self-management, which includes medication adherence, physical activity, and dietary management, is essential for optimal outcomes [2]. Self-management is an active process in which patients understand their conditions, manage symptoms, adhere to prescribed medications, and modify lifestyle habits. These actions are critical for improving health-related quality of life [3]. Digital health technologies have increasingly been applied to support self-management by enabling real-time monitoring, personalized feedback, and behavioral adjustment [4]. Despite variability in effectiveness across modalities and implementation contexts, the adoption of these technologies continues to expand [5,6]. Digital health technologies facilitate the delivery of personalized health information and enable real-time monitoring and feedback through mobile platforms, wearable devices, and remote patient monitoring systems [7,8]. By leveraging digital infrastructures, these technologies facilitate large-scale dissemination of health information across diverse settings, including medically underserved and resource-limited environments, enhancing their relevance to population and public health contexts [7].

However, substantial differences exist in access to digital technologies, digital capabilities, and trust in digital health systems [7,8]. Older adults and socioeconomically disadvantaged populations are more frequently exposed to environments characterized by limited digital health literacy (DHL), restricted access, and reduced trust, thereby increasing the risk of digital exclusion [8]. In a US study of patients with heart failure, more than half of the participants lacked access to mobile health technologies, and limited access was strongly associated with older age, rural residence, and socioeconomic factors [9]. These findings underscore the presence of digital disparities in both access to and effective use of digital health technologies among patients with CVD [9]. Furthermore, the uptake and utilization of digital health technologies are influenced by social determinants of health, including educational attainment, income, race and ethnicity, and sex. DHL has been identified as a key component of digital health equity [10]. Consequently, effective use of digital health technologies is closely associated with DHL, which may determine whether disparities in access translate into successful digitally supported self-management [11].

With the migration of health information delivery to online and mobile platforms, DHL, an extension of traditional health literacy, has emerged as a central construct [12,13]. DHL refers to the ability to access, understand, evaluate, and apply health information obtained in digital environments to support effective health behaviors and improve or maintain health-related quality of life [14,15]. Higher DHL enables individuals to accurately interpret and use digital health information, promoting more effective self-management [16]. Given the complexity and chronicity of CVD, patients require continuous self-management, for which DHL is a critical resource [17].

Studies assessing DHL in CVD have been conducted across diverse settings and have used heterogeneous instruments, most commonly the eHealth Literacy Scale (eHEALS), the Digital Health Literacy Instrument (DHLI), and the eHealth Literacy Questionnaire (eHLQ). These instruments differ in item content and measurement domains depending on their intended purpose and target population and are used to quantify DHL levels [18-20]. In addition, DHL varies across populations according to age, educational attainment, socioeconomic status, and employment status. Older adults and individuals with lower education or income typically report lower DHL, which is associated with reduced use of digital health information and less effective self-management [16,17].

Supporting effective self-management in CVD requires assessment of individual DHL levels, identification of their determinants, and evaluation of associated health outcomes [21]. Despite the widespread availability of mobile apps, remote monitoring tools, and online resources, individuals with low DHL may be unable to use these resources effectively. This limitation increases exposure to inaccurate information and contributes to distrust or avoidance of digital health resources [22]. Emerging evidence suggests that DHL is associated with health behaviors and outcomes in CVD populations; however, quantitative data on DHL levels, determinants, and associations with clinical and patient-reported outcomes remain limited and heterogeneous [21,23].

This systematic review and meta-analysis aimed to assess DHL levels, synthesize factors associated with DHL, and examine DHL-related health outcomes among patients with CVD.

## Methods

### Study Design, Protocol, and Registration

This systematic review and meta-analysis synthesized factors associated with DHL and its health outcomes among patients with CVD. The protocol was registered with PROSPERO (CRD420251068000). Reporting followed the PRISMA (Preferred Reporting Items for Systematic Reviews and Meta-Analyses) 2020 expanded checklist, the PRISMA 2020 (checklist provided in Multimedia Appendix 1) for abstracts checklist [24], and PRISMA-S (PRISMA literature search extension) [25].

Two deviations from the registered protocol were made. First, although the PROSPERO record specified inclusion of English-language publications only, no language restrictions were applied during the final search to minimize potential language bias. Second, studies that focused exclusively on stroke or transient ischemic attack were excluded during full-text screening to enhance clinical homogeneity.

### Eligibility Criteria

Eligibility criteria were based on the participants, intervention or interest, comparisons, outcomes, and study design (PICOS) framework, as follows: adults aged ≥18 years diagnosed with CVD (participants); studies measuring DHL among patients with CVD (intervention or interest); no comparison group was required, as this review did not focus on comparative studies (comparison); determinants of DHL and its health outcomes (outcomes); and quantitative studies and mixed methods studies containing quantitative data were included (study design). Purely qualitative studies and those reporting only DHL scores without examining associated factors or health outcomes were excluded.

For this review, CVD was operationalized as cardiac disease, including coronary artery disease, myocardial infarction, heart failure, arrhythmias, and valvular disease. Primary cerebrovascular disease, such as stroke and transient ischemic attack, was excluded to minimize confounding of DHL assessment by postevent language or cognitive impairment [26]. To capture DHL comprehensively, related constructs including eHealth literacy, electronic health literacy, and mobile health literacy were also considered [14]. The literature search included studies published from 2006 onward, the year in which the concept of eHealth literacy was first introduced [14,27]. No language restrictions were applied. Exclusion criteria were (1) studies not measuring DHL; (2) studies involving children or adolescents; and (3) non–peer-reviewed articles, unpublished works, research protocols, or conference abstracts (Table 1).

**Table 1:** Eligibility criteria based on the PICOS^a^ framework.

Item	Inclusion criteria	Exclusion criteria
Participants	Studies involving adult patients (≥18 years) with CVD^b^	Studies involving children or adolescents (<18 years) or targeting non-CVD populations
Intervention or interest	Studies measuring DHL^c^, including eHealth literacy, electronic health literacy, or mobile health literacy	Studies not measuring DHL or reporting only clinician-reported outcomes
Comparison	Not applicable (no comparator required)	Not applicable
Outcomes	Studies reporting DHL levels, associated factors, or health outcomes related to DHL	Studies not reporting DHL outcomes or related factors
Publication period	Published between January 2006 and January 2026	Published before 2006 or after January 2026
Study designs	Original quantitative studies (cross-sectional, cohort, quasi-experimental, randomized controlled trials, or mixed methods with quantitative outcomes)	Reviews, qualitative-only studies, protocols, conference abstracts, dissertations, editorials, or commentaries

^a^PICOS: participants, intervention or interest, comparison, outcomes, and study design.

^b^CVD: cardiovascular disease.

^c^DHL: digital health literacy.

### Information Sources

A systematic literature search on DHL in patients with CVD was conducted in PubMed (via the National Library of Medicine), Embase (via Elsevier), Cochrane Central Register of Controlled Trials (CENTRAL; via EBSCOhost), CINAHL Plus with Full Text (via EBSCOhost), Scopus (via Elsevier), Web of Science (via Clarivate), and Google Scholar. No additional study registries or online resources were searched. Studies published from 2006 through June 30, 2025, were included. To ensure inclusion of the most recent literature, an updated search was conducted on January 31, 2026, covering the period up to that date. Reference lists of all included studies were manually screened for additional relevant publications; however, no further eligible studies were identified. We contacted the corresponding authors of studies with missing or incomplete data to request additional information; however, no additional data were provided.

### Search Strategy

A combination of MeSH (Medical Subject Headings) terms, free-text keywords, and Boolean operators was used to ensure comprehensive retrieval. The search strategy was independently developed for this study by synthesizing terms from preliminary literature reviews and MeSH databases, without using pre-established search filters or existing search strings. Search terms were developed based on DHL and CVD. In Google Scholar, where Boolean operators and advanced filters are limited, the main keywords (“digital health literacy or eHealth literacy” AND “cardiovascular disease”) were applied. The search strategy was developed in consultation with a librarian and reviewed by the research team prior to implementation. Detailed information on the search process is provided in Multimedia Appendix 2.

### Study Selection

All search records were imported into EndNote 21, and duplicate records were removed electronically. After removing duplicates, the remaining records were exported to Microsoft Excel for screening. During the initial screening of titles and abstracts, the reviewers manually identified and removed any additional duplicates not detected by the software. Two reviewers (EJK and YJS), both experienced in conducting systematic reviews and meta-analyses, independently assessed the records against the inclusion and exclusion criteria and evaluated full texts for eligibility. Discrepancies were resolved through discussion until consensus was reached, and eligible studies were included in the review.

### Data Extraction

Data were extracted on study descriptors (first author, publication year, and country), participant characteristics (sample size, age, and sex), study measures (DHL measurement tools, scale structure and domains, and DHL level), and outcome indicators (factors associated with DHL and related health outcomes). Adjusted estimates for factors associated with DHL and health outcomes were extracted whenever available. When both unadjusted and adjusted results were reported, the final adjusted estimates from multivariable analysis were included. When effect estimates could be derived from reported values, reviewers systematically calculated and extracted them.

### Risk of Bias Assessment

Risk of bias (ROB) and methodological quality were independently assessed by two reviewers (EJK and YJS). The Appraisal Tool for Cross-Sectional Studies (AXIS) was used for cross-sectional studies, the Newcastle-Ottawa Scale (NOS) for cohort or case-control studies, the Revised Cochrane Risk-of-Bias Tool for Randomized Trials (RoB 2), and the Risk of Bias in Nonrandomized Studies of Interventions (ROBINS-I) for nonrandomized interventional studies [28-31]. Disagreements were resolved through consensus.

AXIS comprises 20 items covering study design, sampling, analysis, reporting, and discussion, with each item rated as yes, no, or do not know [28]. NOS evaluates selection, comparability, and outcome, awarding stars within each domain; total scores range from 0 to 9, with higher values indicating better quality [29]. RoB 2 appraises 5 domains: the randomization process, deviations from intended interventions, missing outcome data, measurement of outcomes, and selection of the reported result. Each domain is rated as low risk, some concerns, or high risk. Overall risk is classified as low if all domains are low risk, as some concerns if at least one domain raises concerns but none are high risk, and as high if any domain is high risk or multiple domains raise concerns [30]. ROBINS-I assesses 7 domains: confounding, participant selection, intervention classification, deviations from intended interventions, missing data, outcome measurement, and selection of the reported result. Each domain is graded as low, moderate, serious, or critical ROB, with an overall judgement derived accordingly [31].

### Data Analysis

When at least two studies reported mean eHEALS scores, the means were meta-analyzed using an inverse-variance–weighted random-effects model [32,33]. To avoid double-counting in these analyses, multiple publications from the same cohort were checked, and country-specific studies with clearly defined samples were prioritized to ensure the independence of observations. For assessing determinants and health outcomes of DHL, effect estimates were extracted where reported. Owing to substantial heterogeneity in outcome definitions and analytical approaches, these findings were synthesized narratively.

Random-effects meta-analyses were conducted using the Hartung-Knapp-Sidik-Jonkman method to obtain more robust CIs. This approach accounts for uncertainty in estimating between-study variance and is particularly appropriate when heterogeneity is high or the number of studies is limited [34]. Heterogeneity was evaluated using Cochran *Q* and quantified with Higgins *I*^2^; values of 25%, 50%, and 75% were interpreted as low, moderate, and high heterogeneity, respectively [35]. Although *I*^2^ was reported as a descriptive measure of heterogeneity, it was interpreted with caution because it does not reflect the magnitude of variability in true effects across populations or settings.

Therefore, where applicable, 95% prediction intervals were calculated to quantify between-study heterogeneity in a clinically interpretable manner and to facilitate the real-world interpretation of the pooled estimates. Prediction intervals were derived from the random-effects model, incorporating the estimated between-study variance [36].

Meta-regression was prespecified to explore potential sources of heterogeneity; however, it was not conducted owing to the limited number of included studies [37]. Subgroup analyses were performed to examine variability in mean scores according to three predefined criteria: (1) study region (Asia vs Europe), (2) disease type (heart failure only vs mixed CVD), and (3) mean age (<65 years vs ≥65 years). A leave-one-out sensitivity analysis was performed to assess robustness by examining the contribution of each study to the pooled estimate [38]. All analyses were conducted in R (version 4.4.2; R Foundation for Statistical Computing) using the meta and metafor packages [39-41]. Small-study effects were not assessed in meta-analyses including fewer than 10 studies, as funnel plots and related statistical tests, such as Egger regression, have limited power in analyses with a small number of studies [42,43].

### Certainty of the Evidence

The certainty of evidence for each key outcome was assessed using the Grading of Recommendations Assessment, Development, and Evaluation (GRADE) approach. Assessment domains included ROB, inconsistency, indirectness, imprecision, and publication bias. The GRADE approach was applied to associations between DHL and health outcomes. Based on this assessment, the certainty of evidence for each outcome was classified as high, moderate, low, or very low. Two reviewers (EJK and DYK) independently assessed the certainty of evidence for each outcome, and any disagreements were resolved through consensus [44].

### Ethics Approval

The institutional review board of Chung-Ang University approved the study protocol (1041078-20250603-HR-172).

## Results

### Study Selection

**Figure 1:**
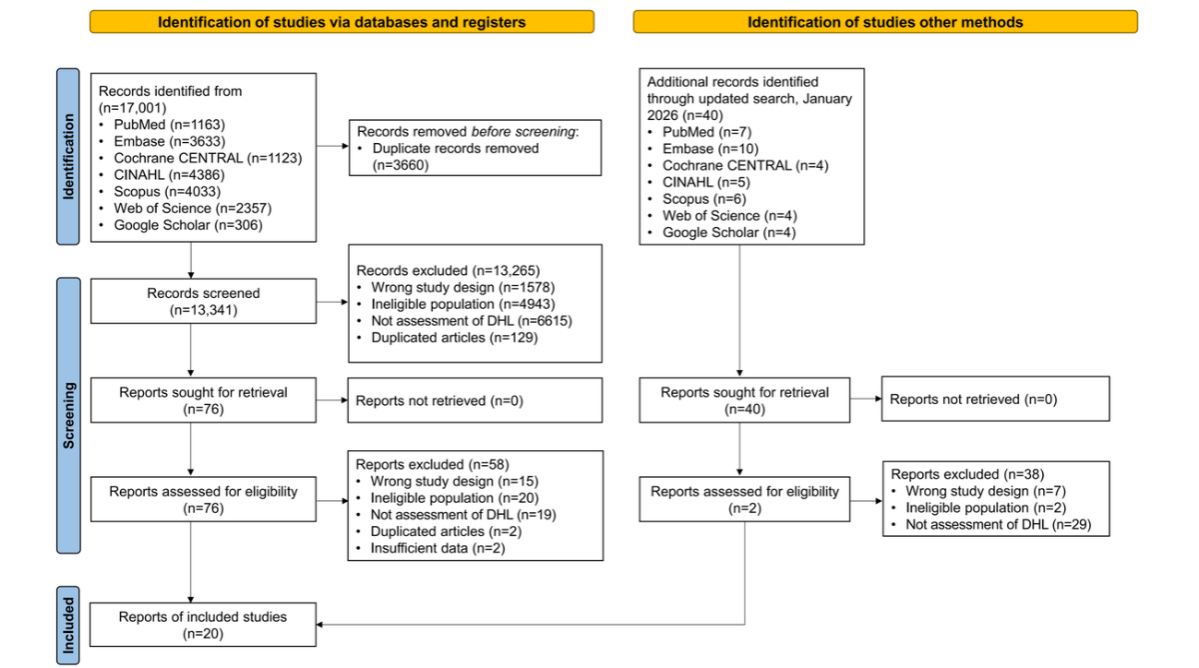
PRISMA (Preferred Reporting Items for Systematic Reviews and Meta-Analyses) flow diagram of study selection. This figure illustrates the process of identification, screening, eligibility assessment, and inclusion of studies in the systematic review in accordance with the PRISMA 2020 guidelines. Records were identified through database searching up to June 30, 2025, with an updated search conducted in January 2026. DHL: digital health literacy.

The initial database search, conducted through June 30, 2025, yielded 17,001 records. Screening and eligibility assessment were performed according to prespecified criteria, and the study selection process is summarized in the PRISMA 2020 flow diagram (Figure 1). After exporting records to EndNote 21 and removing duplicates, 13,341 records remained for title and abstract screening. Of these, 1578 records were excluded owing to incompatible study designs (eg, qualitative studies, protocols, and letters), 4943 records were excluded because participants were not adults with CVD, 6615 records were excluded because DHL was not assessed, and 129 records were removed as remaining duplicates. Full texts of 76 studies were assessed for eligibility, and 58 were excluded owing to ineligible study design (eg, conference abstracts and protocols), ineligible population, absence of DHL assessment, or lack of DHL-related factors or health outcomes. Eighteen studies were initially selected from the primary search. To ensure inclusion of the most recent literature, an updated search was conducted in January 2026, yielding 40 additional records. Following the same screening and eligibility process, 38 records were excluded, and 2 additional studies were included. Consequently, a total of 20 studies met the inclusion criteria and were included in this review (Figure 1).

### Study Characteristics

The 20 studies included in this review were published from 2018 onward, with 14 published after 2023. Most studies used a cross-sectional design (n=11, 55%), followed by 3 quasi-experimental studies and 2 studies each with randomized controlled trial, longitudinal observational, and prospective observational designs. In 5 experimental and cohort studies [45-49], DHL was assessed at multiple time points. The studies were conducted across several countries, predominantly in Europe and Asia.

Heart failure was the most common condition among the study populations (n=10, 50%). Participants included patients who had undergone coronary artery bypass grafting, those with ischemic heart disease, those who had valve surgery or percutaneous coronary intervention, and those with atrial fibrillation, cardiac arrhythmias with a cardiac implantable electronic device, atherosclerosis, or coronary artery disease. The reported mean age of participants ranged from 57.6 to 79.9 years. Most studies recruited adults aged ≥18 years, although some enrolled specific age groups, such as those aged ≥40 years [49], ≥45 years [50], or ≥60 years [51,52]. The total sample size across all studies was 8581 participants, with proportions of males ranging from 49.5% to 83% and females ranging from 17% to 50.5% (Table 2).

**Table 2:** Characteristics of included studies (N=20).

Authors (year)	Study design	Country	Health status	Sample size	Age (years)	Sex (female), n (%)
Melholt et al (2018) [45]	Quasi-experimental	Denmark	CABG^a^, HF^b^, IHD^c^, VS^d^	49	Mean 60.64 (SD 10.75)	9 (18.4)
Chuang et al (2019) [53]	Cross-sectional	Taiwan	HF	141	Mean 65.2 (SD 11.9)	63 (44.7)
Lin et al (2020) [51]	Longitudinal	Iran	HF	468	Mean 69.3 (SD 7.3)	230 (49.2)
Rodríguez Parrado et al (2022) [46]	Quasi-experimental	Colombia	HF	28	Mean 67.3 (SD 12.9)	7 (25.0)
Spindler et al (2022) [47]	Randomized controlled trial	Denmark	HF	137	Intervention group: mean 61.73 (SD 10.75) Control group: mean 61.36 (SD 11.46)	32 (23.4)
Yun et al (2022) [54]	Randomized controlled trial	Spain	HF	178	Median 74	73 (41.0)
Bakhshayesh et al (2023) [55]	Cross-sectional	Iran	HF	200	≤50 years: n=45 (22.5%) >50 years: n=155 (77.5%)	70 (35.0)
Bäuerle et al (2023) [56]	Cross-sectional	Germany	CHF^e^, IHD	290	Mean 57.59 (SD 13.33)	149 (51.4)
Brørs et al (2023) [48]	Prospective	Denmark and Norway	PCI^f^	2924	Mean 66 (SD 11)	582 (19.9)
Ramstad et al (2023) [57]	Prospective	Norway	PCI	1970	Mean 66 (SD 11)	427 (21.7)
Rush et al (2023) [58]	Cross-sectional	Canada	AF^g^	195	Mean 65.36 (SD 10.32)	73 (37.4)
Son et al (2023) [49]	Quasi-experimental	Korea	HF	100	Mean 58.78 (SD 8.83)	17 (17.0)
Vitolo et al (2023) [59]	Cross-sectional	Italy	Arrhythmia, CIED^h^ recipient	300	Median 75 (IQR 66-84)	109 (36.3)
Mohajeri et al (2024) [60]	Cross-sectional	Germany	Atherosclerosis	310	Mean 61.72 (SD 11.44)	130 (41.9)
van Schalkwijk et al (2024) [61]	Longitudinal	Netherlands	Arrhythmia, CHD^i^, HF	305	Mean 64.07 (SD 9.98)	112 (36.7)
Astuti et al (2025) [62]	Cross-sectional	Indonesia	HF	119	Mean 60.18 (SD 13.13)	58 (48.7)
Cuppen et al (2025) [63]	Cross-sectional	Netherlands	HF	61	Mean 79.9 (SD 9.5)	24 (39.3)
Zhao et al (2025) [52]	Cross-sectional	China	CHD	396	Mean 69.78 (SD 6.36)	200 (50.5)
Dibek et al (2025) [64]	Cross-sectional	Turkey	HF	250	≤60 years: n=76 (30.4%) >60 years: n=174 (69.6%)	113 (45.2)
Sun et al (2025) [50]	Cross-sectional	China	CHD	594	45-59 years: n=203 (34.2%) 60-74 years: n=264 (44.4%) ≥75 years: n=127 (21.4%)	235 (42.6)

^a^CABG: coronary artery bypass grafting.

^b^HF: heart failure.

^c^IHD: ischemic heart disease.

^d^VS: valve surgery.

^e^CHF: congestive heart failure.

^f^PCI: percutaneous coronary intervention.

^g^AF: atrial fibrillation.

^h^CIED: cardiac implantable electronic device.

^i^CHD: coronary heart disease.

The most frequently used instrument for assessing DHL was the eHEALS [18] (n=11), followed by the DHLI [19] (n=2) and the eHLQ [20] (n=1). The Digital Health Readiness Questionnaire (DHRQ) [65] was used in one study (n=1). Because newer DHL instruments, such as the eHLQ and DHRQ, incorporate attitudinal components (eg, motivation and attitudes) into the construct of DHL, some studies additionally assessed digital-related attitudes, such as internet anxiety and digital confidence [56,60]. One study used a self-developed instrument [46] (Table 3; Multimedia Appendix 3 [36-48,51,53-56]). In studies with incomplete or unreported essential data [55,56,60], the corresponding authors were contacted to request additional information; however, no responses were received.

**Table 3:** Digital health literacy measurement tools and levels of the included studies.

Authors (year)	DHL^a^ measurement	DHL level
Melholt et al (2018) [45]	eHEALS^b^	Mean 28.80 (SD 5.96)^c^ (baseline)
Chuang et al (2019) [53]	eHEALS	Mean 26.2 (SD 5.7)
Lin et al (2020) [51]	eHEALS	Mean 28.16 (SD 5.46)
Rodríguez Parrado et al (2022) [46]	Researcher-developed questionnaire	Mean 2.33 (SD 1.25)^c^ (baseline)
Spindler et al (2022) [47]	eHLQ^d^	Intervention group: mean 2.98 (SD 3.22) Control group: mean 2.71 (SD 3.17) (after 6 months)^e^
Yun et al (2022) [54]	Researcher-developed questionnaire	72% lower ICT^f^ skills (n=128) 28% middle or higher ICT skills (n=50)
Bakhshayesh et al (2023) [55]	eHEALS	Mean 18.09 (SD 9.08)
Bäuerle et al (2023) [56]	Internet anxiety, digital confidence, prior experiences with mobile health interventions	Not reported
Brørs et al (2023) [48]	eHEALS	Mean 27.27 (SD 6.28) (baseline)
Ramstad et al (2023) [57]	eHEALS	Mean 25.71 (SD 6.22) (baseline)
Rush et al (2023) [58]	30-item composite tool	Computer self-efficacy: mean 5.38 (SD 1.27) Health technology self-efficacy: mean 5.63 (SD 1.01) Attitude toward health technology: mean 5.46 (SD 0.87)
Son et al (2023) [49]	eHEALS	Intervention group: mean 24.32 (SD 9.55) Control group: mean 25.58 (SD 9.79) (baseline)
Vitolo et al (2023) [59]	DHLI^g^	Mean 48.58 (SD 24.16)
Mohajeri et al (2024) [60]	Internet anxiety, digital confidence, digital overload	Internet anxiety: mean 1.58 (SD 0.80) Digital confidence: mean 3.87 (SD 0.98) Digital overload: mean 1.94 (SD 1.01)
van Schalkwijk et al (2024) [61]	DHLI	Mean 2.97 (SD 0.60)
Astuti et al (2025) [62]	eHEALS	Mean 26.35 (SD 7.40)
Cuppen et al (2025) [63]	DHRQ^h^	Mean 38.4 (SD 17.7)
Zhao et al (2025) [52]	eHEALS	Mean 18.18 (SD 10.11)
Dibek et al (2025) [64]	eHEALS	Mean 21.9 (SD 9.7)
Sun et al (2025) [50]	eHEALS	Median 21 (IQR 13-29)

^a^DHL: digital health literacy.

^b^eHEALS: eHealth Literacy Scale.

^c^Scores calculated by authors.

^d^eHLQ: eHealth Literacy Questionnaire.

^e^Baseline data were not reported in this study.

^f^ICT: information and communications technology.

^g^DHLI: Digital Health Literacy Instrument.

^h^DHRQ: Digital Health Readiness Questionnaire.

### Quality Appraisal

The included studies comprised 11 cross-sectional, 2 longitudinal observational, 2 prospective observational, 2 randomized controlled trials, and 3 quasi-experimental studies. ROB and methodological quality were appraised using tools appropriate for each study design, and a summary of these assessments is presented in Table 4.

**Table 4:** Summary of quality assessment of the included studies^a^.

Appraisal tool and study design				Overall quality assessment
**AXIS^b^**				
	**Cross-sectional**			
		Chuang et al (2019) [53]	18/20	
		Bakhshayesh et al (2023) [55]	17/20	
		Bäuerle et al (2023) [56]	17/20	
		Rush et al (2023) [58]	18/20	
		Vitolo et al (2023) [59]	16/20	
		Mohajeri et al (2024) [60]	18/20	
		Astuti et al (2025) [62]	17/20	
		Cuppen et al (2025) [63]	16/20	
		Zhao et al (2025) [52]	18/20	
		Dibek et al (2025) [64]	17/20	
		Sun et al (2025) [50]	17/20	
**NOS^c^**				
	**Longitudinal/prospective**			
		Lin et al (2020) [51]	7/9	
		Brørs et al (2023) [48]	6/9	
		Ramstad et al (2023) [57]	5/9	
		van Schalkwijk et al (2024) [61]	5/9	
**RoB 2^d^**				
	**Randomized controlled trial**			
		Spindler et al (2022) [47]	High risk	
		Yun et al (2022) [54]	Some concerns	
**ROBINS-I^e^**				
	**Quasi-experimental**			
		Melholt et al (2018) [45]	Moderate	
		Rodríguez Parrado et al (2022) [46]	Moderate	
		Son et al (2023) [49]	Moderate	

^a^Methodological quality was assessed using validated appraisal tools appropriate for each study design. Overall quality ratings or scores are reported as provided by each appraisal framework.

^b^AXIS: Appraisal Tool for Cross-Sectional Studies.

^c^NOS: Newcastle-Ottawa Quality Assessment Scale.

^d^RoB 2: Revised Cochrane Risk-of-Bias Tool for Randomized Trials.

^e^ROBINS-I: Risk of Bias in Nonrandomized Studies of Interventions.

The 11 cross-sectional studies were evaluated using the AXIS tool, with quality scores ranging from 16 to 18 out of a maximum of 20 points. However, most studies did not report information on nonresponders or provide response rates. In addition, characteristics of nonresponders or comparisons with responders were not reported (Multimedia Appendix 4 [50,52,53,55,56,58-60,62-64]). The 4 cohort studies (longitudinal, prospective observational studies) were assessed using the NOS, with scores ranging from 5 to 7 out of 9. According to the Agency for Healthcare Research and Quality (AHRQ) thresholds, 2 studies were rated as good quality and 2 as poor quality. The studies rated as poor quality relied on self-reported outcomes and lacked clear reporting of follow-up rates or attrition (Multimedia Appendix 5 [48,51,57,61]).

The 2 randomized controlled trials were appraised using RoB 2 and were rated as having “some concerns” and “high ROB,” respectively. In both studies, the randomization process and blinding procedures were not clearly reported, and insufficient information was available to support a low risk in these domains. Furthermore, neither study provided adequate details regarding missing outcome data (Multimedia Appendix 6 [47,54]). The 3 quasi-experimental studies were evaluated using ROBINS-I and were all judged to have a moderate ROB. Concerns primarily arose in the measurement of the outcomes domain, where outcome assessors may have been aware of participants’ intervention status (Multimedia Appendix 7 [45,46,49]).

### Factors Associated With DHL

Across the 20 included studies, factors associated with DHL were categorized into 5 domains: demographic (age, sex, education, and residence), clinical (cognitive function, laboratory findings, and disease severity), physical health (functional status and frailty), psychosocial (social support and caregiving needs), and digital technology use (prior experience) (Table 5; Multimedia Appendix 8 [45-49,51-63]). Lower educational attainment (n=6) and older age (n=5) were most frequently associated with lower DHL. Other factors included female sex (n=2), disease severity (n=2), lower or higher social support (n=2), and limited prior experience with digital technologies (n=2).

**Table 5:** Factors associated with digital health literacy of the included studies.

Category/subcategory			Significant factors^a^		References
**Demographic variables**					
	Age	Older age		[49,50,54,55,64]	
	Sex	Female		[54,58]	
	Education	Lower educational attainment		[45,50,54-56,64]	
	Marital status	Married status		[64]	
	Economic status	Lower economic status		[64]	
	Employment status	Unemployment		[64]	
	Residence	Rural residence		[56]	
**Clinical variables**					
	Cognitive function	Lower cognitive function		[54]	
	Laboratory findings	Lower BMI, DBP^b^, Hb^c^, NT-proBNP^d^		[54]	
	Diagnosis duration	>6 years		[64]	
	Disease severity	Higher NYHA^e^ (or HF stage) functional class		[54,64]	
	Prior cardiac intervention	Absence of PCI or CABG		[50]	
**Physical health**					
	Functional status	Greater functional dependence		[54]	
	Frailty	Higher frailty status		[59]	
	Smoking status	Smokers		[64]	
**Psychosocial variables**					
	Health behaviors	Lower health behaviors		[64]	
	Social support	Higher social support		[54]	
	Social support	Lower social support		[52]	
	Caregiving needs	Greater caregiver needs		[54]	
**Digital technology use**					
	Prior experience with digital technology	Low use of digital tools (computer, internet, national health portal)		[45,57]	

^a^All listed factors were negatively associated with digital health literacy.

^b^DBP: diastolic blood pressure.

^c^Hb: hemoglobin.

^d^NT-proBNP: N-terminal pro-B-type natriuretic peptide.

^e^NYHA: New York Heart Association classification of heart failure functional status.

### Association Between DHL and Health Outcomes

Health outcomes associated with DHL among adults with CVD were categorized as cognitive outcomes (disease knowledge and empowerment), health behaviors (smoking, self-care behaviors, physical activity, and medication adherence), clinical outcomes (cardiac events), health-related outcomes (depression, anxiety, and quality of life), and digital health-related outcomes (use of digital health technology and acceptance of digital health; Table 6).

Across the included studies, higher DHL was most frequently associated with digital health–related outcomes, particularly greater use and higher acceptance of digital health technologies (n=8). Other reported associations included improved self-care behaviors (n=3) and enhanced quality of life (n=2).

Most studies reported their results using regression analysis, while others used correlation analysis, logistic regression, or Cox regression for survival analyses. Effect sizes were reported in various forms depending on study design, including standardized beta coefficient (*β*), unstandardized coefficient (*B*), odds ratio, correlation coefficient (*r*), and hazard ratio (Multimedia Appendix 8).

**Table 6:** Association between digital health literacy and health outcomes.

Category, subcategory, and observed health outcomes			References	Certainty^a^
**Cognitive outcomes**				
	**Disease knowledge**			
		Greater HF^b^ knowledge	[53]	⊕OOO
	**Empowerment**			
		Increased empowerment	[46]	⊕OOO
**Health behaviors**				
	**Smoking**			
		Lower likelihood of smoking	[48]	⊕OOO
	**Self-care behaviors**			
		Higher self-care behaviors	[53,55,62]	⊕OOO
	**Physical activity**			
		Higher physical activity	[48]	⊕OOO
	**Medication adherence**			
		Improved medication adherence	[51]	⊕OOO
**Clinical outcomes**				
	**Cardiac events**			
		Lower risk of cardiac events	[51]	⊕OOO
	**Symptom status**			
		Lower symptom burden	[64]	⊕OOO
**Health-related outcomes**				
	**Depression**			
		Reduced depressive symptoms	[48]	⊕OOO
	**Anxiety**			
		Reduced anxiety symptoms	[48]	⊕OOO
	**Quality of life**			
		Improved quality of life	[51,55]	⊕OOO
**Digital health-related outcomes**				
	**Use of digital health technology**			
		Greater use of the internet, mobile apps, and electronic health applications	[47,57,61]	⊕⊕OO^c^ ⊕OOO^d^
		Higher technology self-efficacy and motivation; lower technophobia	[52]	⊕OOO
	**Acceptance of digital health**			
		Increased acceptance of mobile health; greater willingness to participate in telemonitoring	[56,60,63]	⊕OOO
		Higher telehealth satisfaction and more positive attitudes toward health care technology; lower telehealth satisfaction with higher computer self-efficacy	[58]	⊕OOO

^a^The certainty of evidence for each outcome was evaluated using the GRADE (Grading of Recommendations Assessment, Development, and Evaluation) approach: ⊕⊕⊕⊕ indicates high certainty, ⊕⊕⊕O moderate certainty, ⊕⊕OO low certainty, and ⊕OOO very low certainty.

^b^HF: heart failure.

^c^Certainty of evidence based on randomized controlled trial evidence.

^d^Certainty of evidence based on observational studies.

### Meta-Analysis Results

**Figure 2:**
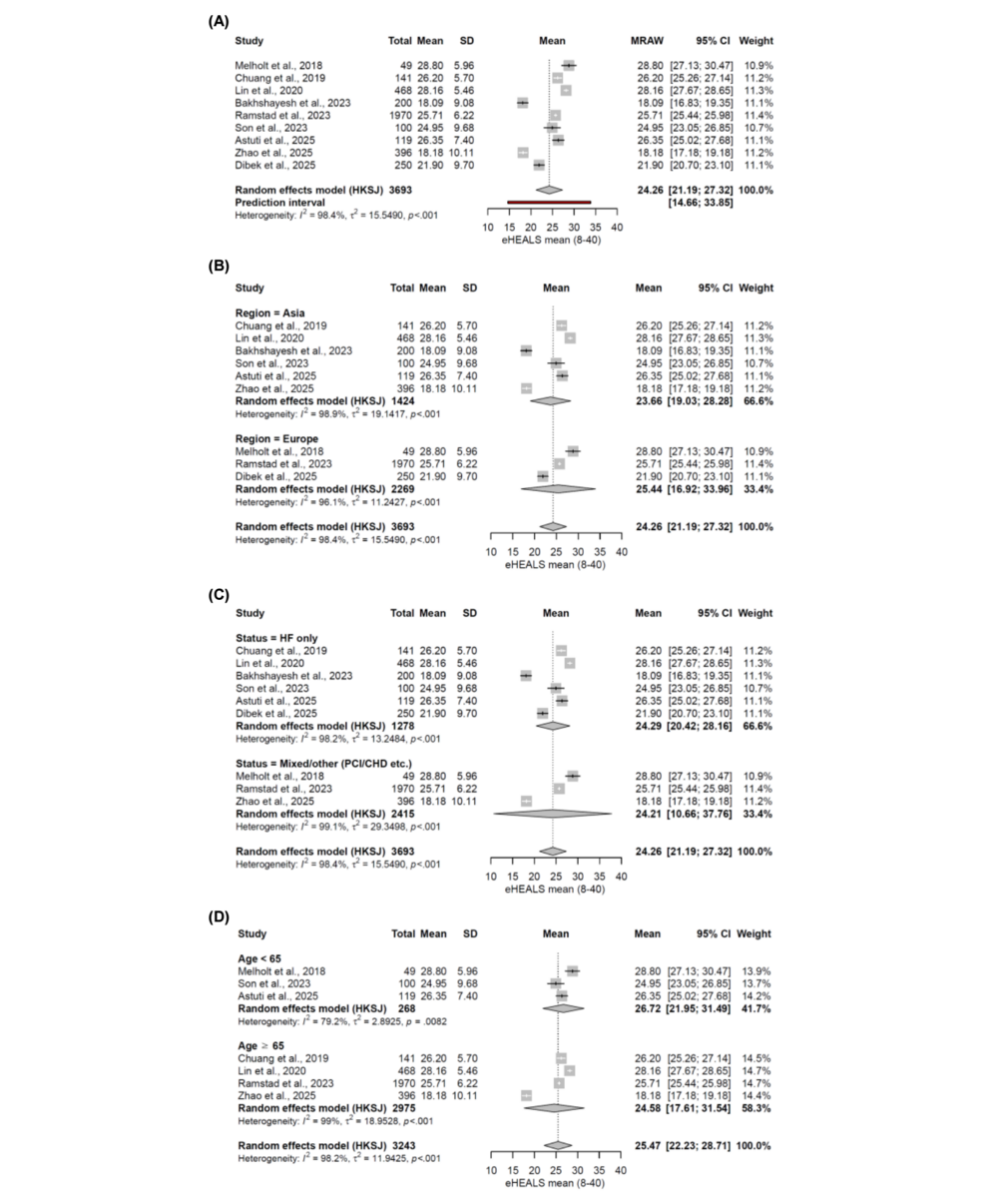
Forest plot of pooled mean eHEALS scores across included studies: (A) overall pooled mean eHEALS score, (B) subgroup meta-analysis by study region (Asia vs Europe), (C) subgroup meta-analysis by disease type (heart failure only vs mixed CVD conditions, including percutaneous coronary intervention or coronary heart disease), and (D) subgroup meta-analysis by mean age group (<65 years vs ≥65 years). This figure summarizes results from 9 studies published between 2018 and 2025, including 5 cross-sectional studies, 2 quasi-experimental studies, 1 longitudinal study, and 1 prospective cohort study. Study populations consisted of adult patients with heart failure or other cardiovascular diseases, recruited across Asia (Taiwan, Iran, South Korea, Indonesia, and China) and Europe (Denmark, Norway, and Turkey). Sample sizes ranged from 49 to 1970 participants per study, with a total of 3693 participants included in the overall meta-analysis [45-47,51,54,56,60,62,63]. eHEALS: eHealth Literacy Scale; HKSJ: Hartung-Knapp-Sidik-Jonkman method; MRAW: mean raw; PCI/CHD: percutaneous coronary intervention/coronary heart disease.

Nine studies reported mean eHEALS scores, which were pooled using a random-effects model. The overall pooled mean eHEALS score was 24.26 (95% CI 21.19-27.32; *P*<.001; *I*^2^=98.4%; *τ*^2^=15.55; *τ*=3.94). The 95% prediction interval ranged from 14.66 to 33.85, indicating substantial variability in true eHEALS scores across populations and settings. Subgroup analyses were conducted by region (n=9), disease type (n=9), and mean age (n=7). Studies conducted in Europe reported slightly higher eHEALS scores than those conducted in Asia. Studies with a mean age <65 years exhibited higher scores than those with a mean age ≥65 years. Studies that included only patients with heart failure demonstrated eHEALS scores nearly identical to those reported in studies of mixed CVD populations. No statistically significant subgroup differences were observed in analyses stratified by region (*P*=.50), disease type (*P*=.98), or age (*P*=.38; Figure 2). Leave-one-out sensitivity analysis indicated that the pooled eHEALS score ranged from 23.70 to 25.02, with a maximum variation of 1.32 points across iterations.

### Assessment of Reporting Biases

Assessment of reporting biases was not conducted because fewer than 10 studies were included in the meta-analysis, as described in the Methods section.

### Certainty of Evidence

The certainty of evidence for each outcome, as assessed using the GRADE framework, was rated as low or very low (Table 6). The evidence was initially low, reflecting its primary reliance on observational studies. Certainty was further downgraded for imprecision, attributable to the limited number of studies and wide CIs. Detailed ratings and the rationale for downgrades are provided in Multimedia Appendix 9.

## Discussion

### Principal Findings

This review synthesized evidence on DHL levels, factors associated with DHL, and related health outcomes among adults with CVD, a population for whom sustained self-management is critical. Overall, the findings indicate that DHL levels among adults with CVD are relatively low and characterized by substantial between-study heterogeneity. The wide prediction interval further reflects this heterogeneity, indicating that DHL levels may vary considerably across clinical contexts even when the pooled mean estimate is similar [36]. Sociodemographic characteristics, particularly older age, lower educational attainment, and female sex, were commonly associated with lower DHL. Higher DHL was associated with more favorable health outcomes, including improved self-care behaviors, better quality of life, and greater acceptance of digital health technologies. Collectively, these findings provide an empirical overview of the role of DHL in supporting self-management in patients with CVD.

### DHL Levels Across Studies

DHL is increasingly recognized as a key determinant of both public and individual health [66]. Using the commonly applied cutoff of 26 on the eHEALS to indicate limited DHL [67], the pooled eHEALS score identified in this review was below this threshold, suggesting generally low DHL levels among adults with CVD. Compared with findings from a previous systematic review of individuals with chronic diseases, including diabetes mellitus and hypertension [37], DHL levels observed in the present review were comparatively lower. According to the American Heart Association, the prevalence of CVD increases substantially with advancing age, with the highest burden observed among older adults [68]. Accordingly, the relatively low DHL levels identified in this review should be interpreted in the context of the older age distribution of CVD populations. This finding is consistent with a previous meta-analysis by Kanejima et al [26], which reported a high prevalence of limited health literacy among patients with CVD. Effective management of CVD requires adherence to medication regimens, dietary modifications, and timely symptom management and response [12]. These tasks increasingly depend on accessing, interpreting, and applying health information delivered through digital platforms [69]. The cognitive and technological demands associated with CVD management may contribute to challenges in engaging with digital health information and may partially explain the lower observed DHL levels observed in this population [70].

Subgroup analyses were limited to variables consistently reported across studies, including region, disease type, and mean age. No statistically significant subgroup differences were identified, which may reflect limited statistical power due to the small number of included studies [71]. Nevertheless, directional trends warrant consideration. Studies conducted in Europe tended to report higher DHL levels than those conducted in Asia, potentially reflecting differences in digital health infrastructure, access to health technologies, and system-level support [72]. In addition, higher DHL levels observed among participants younger than 65 years suggest that age-related disparities in DHL persist within CVD. The CIs around the pooled estimate reflect uncertainty in the average DHL level across studies and should be interpreted with caution, given the substantial between-study heterogeneity. Moreover, the wide prediction interval indicates considerable variability across settings [36]. This variability suggests that DHL proficiency is not uniformly low among adults with CVD but is likely influenced by contextual and system-level factors [73].

### Determinants and Health Outcomes of DHL

The factors identified in this review suggest that DHL among adults with CVD is shaped by both individual characteristics and broader social and digital contexts. Notably, the included studies predominantly focused on individual-level digital experiences, competencies, and perceptions, rather than structural or system-level digital environments. In addition, older age and lower educational attainment were consistently associated with lower DHL in adults with CVD. According to the 2026 American Heart Association Heart Disease and Stroke Statistics, CVD and heart failure disproportionately affect older adults [68]. Furthermore, large-scale cohort studies consistently demonstrate that lower educational attainment is associated with an increased risk of CVD [74]. Collectively, these sociodemographic characteristics provide critical context for interpreting DHL levels in patients with CVD. In this review, additional factors frequently associated with lower DHL included female sex, rural residence, poorer self-rated health, lower social support, and limited prior experience with digital technologies. These findings align with a recent meta-analysis of 17 studies in mixed populations, which identified age, ethnicity, income, employment status, education, and health status as key correlates of eHealth literacy [75].

However, rather than framing these demographic characteristics solely as individual deficits, they should be interpreted through the lens of Digital Determinants of Health [76]. According to the American Heart Association, Digital Determinants of Health—which encompass DHL, digital infrastructure, access, and digital inequity—play a pivotal role in shaping cardiovascular outcomes [77]. Most studies included in this review treated DHL as an individual-level attribute, emphasizing personal skills and behaviors. This approach reflects a broader tendency in the literature to frame DHL as an individual responsibility, with limited consideration of health system structures, provider practices, or service-level contexts that may influence digital engagement [76]. From this perspective, disparities in DHL may not be fully explained by individual-level characteristics alone, highlighting the potential relevance of structural and provider-level factors [76,77].

Health outcomes associated with DHL clustered into 5 domains: cognitive, health behavior, clinical, health-related, and digital health–related outcomes. Across these domains, higher DHL was most consistently associated with improvements in cognitive understanding and health behaviors, including greater disease knowledge, increased empowerment, and more effective self-care practices such as medication adherence and physical activity. These findings suggest that DHL may facilitate self-management by enhancing information processing and supporting the translation of health information into everyday behaviors [14]. Digital health–related outcomes were most consistently associated with DHL in this review. Higher DHL was associated with greater acceptance and use of digital health technologies, stronger technology self-efficacy, and lower technophobia. These digital indicators appear closely aligned with the functional attributes of DHL, conceptualized as the ability to seek, appraise, and apply health information in digital contexts [14,27]. Therefore, DHL functions as a foundational competency that underpins a range of outcomes related to the use of digital health tools, supporting sustained self-management [22].

In this review, evidence linking DHL to clinical outcomes was comparatively limited. While some included studies reported associations between higher DHL and lower risks of cardiac events [51] or improved symptom status [64], these findings were based on a small number of studies and heterogeneous outcome measures. Further longitudinal research is required to elucidate the pathways linking DHL to hard clinical outcomes, including studies that integrate patient-reported perspectives on providers and system-level digital support.

The overall certainty of these findings is limited by study design characteristics and ROB across the included studies. Most synthesized studies were observational and therefore inherently susceptible to confounding. In addition, quality assessment revealed methodological limitations, particularly incomplete reporting of participant flow, nonresponders, and attrition. Given that DHL is influenced by sociodemographic factors such as age, educational attainment, and socioeconomic status [20], insufficient information on sample selection and follow-up may introduce selection bias and affect the interpretation of DHL estimates.

As digital health environments continue to evolve rapidly, transparent reporting of participant characteristics and study procedures is increasingly critical for contextualizing findings [78]. Improving reporting of sample representativeness and participant flow would enhance the interpretability of future studies and contribute to a more robust evidence base on DHL among adults with CVD. Considering the observed heterogeneity, ROB, and limitations identified in the GRADE framework, the overall certainty of evidence regarding associations between DHL and health outcomes was rated as low. Consequently, these findings should be interpreted as indicative of broad trends rather than a precise estimate applicable to all individual patients.

### Implications for Clinical Practice and Health Policy

These findings have key implications for clinical practice. DHL and digital access should be considered central determinants of health equity that influence patients’ capacity for effective self-management, rather than merely reflecting individual-level limitations [73]. The emphasis on individual-level factors in the existing literature underscores the need to also consider the health care system and provider-level influences on patients’ digital engagement [76]. Accordingly, health care systems and providers may play a critical role in supporting equitable self-care by addressing digital barriers [77].

In clinical settings, assessing patients’ digital access and competencies as part of routine assessment—alongside other social and contextual factors—may help identify individuals at risk of digital exclusion [73]. These assessments should not be treated solely as screening tools, but as a foundation for adapting service delivery and provider practices to accommodate varying levels of digital readiness [76]. For patients with low DHL or limited access, the mere provision of digital technologies may be insufficient. System-level strategies—such as supported-use models integrated into routine care and service designs that enhance accessibility—may be required, alongside patient education programs or involvement of family caregivers where appropriate [69,73,77]. Adopting a digital inclusion–oriented approach may help ensure that digital health technologies act as facilitators rather than barriers to care, thereby supporting more effective and sustained self-management among adults with CVD [69,77].

### Strengths and Limitations

This review has several strengths. First, to our knowledge, it is the first systematic review to comprehensively synthesize health outcomes associated with DHL in patients with CVD using a multidimensional framework encompassing 5 distinct domains. Second, by incorporating findings from multidimensional instruments such as the DHLI [19], eHLQ [20], and DHRQ [65], this study aligns with the evolving conceptual definition of DHL [14]. This approach extends beyond traditional eHealth literacy and captures both functional skills and attitudinal components (eg, motivation and digital confidence) required in contemporary digital health environments.

Several limitations warrant consideration. First, most included studies were cross-sectional and showed substantial variability in analytic methods and confounder adjustment, limiting comparability. Second, evidence was limited for psychosocial determinants as well as objective clinical endpoints and biological markers. Many biomarkers and clinical events were evaluated in only a small number of studies, often from single-center samples with modest sizes, which constrains generalizability. Third, reporting of nonresponders and participant flow was often incomplete. This limited transparency may compromise assessments of sample representativeness and increase the risk of selection bias, particularly given known sociodemographic gradients in DHL [21]. Finally, substantial between-study heterogeneity was observed across study populations, settings, measurement instruments, and analytic approaches. Accordingly, pooled estimates and prediction intervals should be interpreted with caution.

### Conclusions

To our knowledge, this review is one of the first to integrate evidence on DHL levels, associated factors, and DHL-related health outcomes among adults with CVD. Overall, DHL varied widely and showed consistent gradients by age and socioeconomic disadvantage, with lower DHL generally associated with poorer engagement with digital health resources and less favorable self-management–related outcomes. Although the pooled mean eHEALS score indicates modest DHL on average, the wide prediction interval indicates considerable variability across populations and clinical settings. By synthesizing DHL levels, determinants, and health outcomes in CVD populations, this review offers an integrated evidence base that extends beyond single-instrument assessments (eg, eHEALS) and supports interpretation of DHL in relation to structural and health system contexts.

Our findings highlight the need for routine assessment of DHL and targeted support for patients with limited DHL, as well as the importance of designing and delivering digital tools that are accessible and easy to use in routine CVD care. Future studies using longitudinal and interventional designs are needed to clarify causal pathways linking DHL to self-management and health outcomes, and to examine how provider- and health system–level factors shape and support DHL in routine care.

## Appendix

Multimedia Appendix 1 PRISMA 2020 reporting checklists (PRISMA 2020, PRISMA Abstract, and PRISMA-S).

Multimedia Appendix 2 Literature search strategy.

Multimedia Appendix 3 Summary of digital health literacy measurement tools included in this review.

Multimedia Appendix 4 Quality assessment for cross-sectional studies using the AXIS (Appraisal Tool for Cross-Sectional Studies) tool.

Multimedia Appendix 5 Newcastle–Ottawa Quality Assessment of longitudinal observational and prospective observational studies.

Multimedia Appendix 6 Risk-of-bias tool for randomized controlled trials.

Multimedia Appendix 7 Risk of bias in nonrandomized studies of interventions.

Multimedia Appendix 8 Study-level digital health literacy levels, associated factors, and health outcomes.

Multimedia Appendix 9 GRADE (Grading of Recommendations Assessment, Development, and Evaluation) assessment.

## Abbreviations

AHRQ: Agency for Healthcare Research and Quality
AXIS: Appraisal Tool for Cross-Sectional Studies
CVD: cardiovascular disease
DHL: digital health literacy
DHLI: Digital Health Literacy Instrument
DHRQ: Digital Health Readiness Questionnaire
eHEALS: eHealth Literacy Scale
eHLQ: eHealth Literacy Questionnaire
GRADE: Grading of Recommendations Assessment, Development, and Evaluation
MeSH: Medical Subject Headings
NOS: Newcastle-Ottawa Scale
PICOS: participants, intervention or interest, comparisons, outcomes, and study design
PRISMA: Preferred Reporting Items for Systematic Reviews and Meta-Analyses
PRISMA-S: Preferred Reporting Items for Systematic Reviews and Meta-Analyses literature search extension
ROB: risk of bias
RoB 2: Revised Cochrane Risk-of-Bias Tool for Randomized Trials
ROBINS-I: Risk of Bias in Nonrandomized Studies of Interventions

## Data Availability

The data underlying the findings of this study are available from the corresponding authors upon reasonable request.
