# Supplementary material for: Determinants and Health Outcomes of Digital Health Literacy in Patients With Cardiovascular Disease: Systematic Review and Meta-Analysis
Source: J Med Internet Res. 2026 Mar 24;28:e89102. doi: 10.2196/89102 (PMC13058533; doi:10.2196/89102)
Supplement: Multimedia Appendix 2 [file jmir_v28i1e89102_app2.docx]

**Literature Search Strategy**

| **Database** | **Search no** | **Search strategy** | **Articles retrieved** |
| --- | --- | --- | --- |
| PubMed | #1 | ("digital health"[Mesh] OR "telemedicine"[Mesh]) OR  ("digital health literacy"[tiab] OR "eHealth literacy"[tiab] OR "digital literacy"[tiab] OR "electronic health literacy"[tiab] OR "mobile health literacy"[tiab] OR "digital health"[tiab] OR "telemedicine"[tiab] OR "eHealth"[tiab] OR "digital health technology"[tiab] OR "medical technology"[tiab]) | 90.545 |
|  | #2 | ("cardiovascular disease"[MeSH] OR "coronary disease"[MeSH] OR "coronary artery disease"[MeSH] OR "angina pectoris"[MeSH] OR "myocardial infarction"[MeSH] OR "heart failure"[MeSH] OR "arrhythmias, cardiac"[MeSH] OR "atrial fibrillation"[MeSH] OR "heart arrest"[MeSH] OR "peripheral arterial disease"[MeSH] OR "rheumatic heart disease"[MeSH] OR "heart valve diseases"[MeSH])  OR ("cardiovascular disease"[tiab] OR "coronary disease"[tiab] OR "coronary artery disease"[tiab] OR "angina pectoris"[tiab] OR "angina"[tiab] OR "ischemic heart disease"[tiab] OR "myocardial infarction"[tiab] OR “heart infarction”[tiab] OR "heart failure"[tiab] OR "congestive heart failure"[tiab] OR "arrhythmias, cardiac"[tiab] "atrial fibrillation"[tiab] OR "cardiac arrhythmia"[tiab] OR "heart arrhythmia[tiab]" OR "arrhythmia"[tiab] OR "ventricular arrhythmia"[tiab] OR "heart arrest"[tiab] OR "cardiac arrest"[tiab] OR "sudden cardiac death"[tiab] OR "peripheral arterial disease"[tiab] OR "intermittent claudication"[tiab] OR "critical limb ischemia"[tiab] OR "peripheral artery stenosis"[tiab] OR "rheumatic heart disease"[tiab] OR "heart valve diseases"[tiab] OR "mitral valve stenosis"[tiab] OR "aortic valve disease"[tiab] OR "multivalvular disease"[tiab] OR "valvular heart disease"[tiab]) | 1,046,148 |
|  | #3 | #1 and #2 Filters: Free full text, Full text, English, Humans, from 2006/1/1 - 2025/6/30 | 1,163 |
| EMBASE | #1 | ('digital health'/exp OR 'digital health technology'/exp OR 'medical technology'/exp) OR  ('digital health literacy':ab,ti OR 'eHealth literacy':ab,ti OR 'digital literacy':ab,ti OR 'electronic health literacy':ab,ti OR 'mobile health literacy':ab,ti OR 'digital health':ab,ti OR 'telemedicine':ab,ti OR 'eHealth':ab,ti OR 'digital health technology':ab,ti OR 'medical technology':ab,ti) | 104,885 |
|  | #2 | ('cardiovascular disease'/exp OR 'coronary artery disease'/exp OR 'angina pectoris'/exp OR 'heart infarction'/exp OR 'heart failure'/exp OR 'atrial fibrillation'/exp OR 'heart arrhythmia'/exp OR 'heart arrest'/exp OR 'peripheral arterial disease'/exp OR 'rheumatic heart disease'/exp OR 'valvular heart disease'/exp) OR ('cardiovascular disease':ab,ti OR 'coronary disease':ab,ti OR 'coronary artery disease':ab,ti OR 'angina pectoris':ab,ti OR 'angina':ab,ti OR 'ischemic heart disease':ab,ti OR 'myocardial infarction':ab,ti OR 'heart infarction':ab,ti OR 'heart failure':ab,ti OR 'congestive heart failure':ab,ti OR 'arrhythmias, cardiac':ab,ti OR 'atrial fibrillation':ab,ti OR 'cardiac arrhythmia':ab,ti OR 'heart arrhythmia':ab,ti OR 'arrhythmia':ab,ti OR 'ventricular arrhythmia':ab,ti OR 'heart arrest':ab,ti OR 'cardiac arrest':ab,ti OR 'sudden cardiac death':ab,ti OR 'peripheral arterial disease':ab,ti OR 'intermittent claudication':ab,ti OR 'critical limb ischemia':ab,ti OR 'peripheral artery stenosis':ab,ti OR 'rheumatic heart disease':ab,ti OR 'heart valve diseases':ab,ti OR 'mitral valve stenosis':ab,ti OR 'aortic valve disease':ab,ti OR 'multivalvular disease':ab,ti OR 'valvular heart disease':ab,ti) | 6,278,580 |
|  | #3 | #1 AND #2 AND 'article'/it AND [english]/lim AND [humans]/lim AND [2006-2025]/py | 3,633 |
| Cochrane CENTRAL | #1 | ("digital health literacy" OR "eHealth literacy" OR "digital literacy" OR "electronic health literacy" OR "mobile health literacy" OR "digital health" OR "telemedicine" OR "eHealth" OR "digital health technology" OR "medical technology") | 10,764 |
|  | #2 | ("cardiovascular disease" OR "coronary disease" OR "coronary artery disease" OR "angina pectoris" OR "angina" OR "ischemic heart disease" OR "myocardial infarction" OR "heart infarction" OR "heart failure" OR "congestive heart failure" OR "arrhythmias, cardiac" OR "atrial fibrillation" OR "cardiac arrhythmia" OR "heart arrhythmia" OR "arrhythmia" OR "ventricular arrhythmia" OR "heart arrest" OR "cardiac arrest" OR "sudden cardiac death" OR "peripheral arterial disease" OR "intermittent claudication" OR "critical limb ischemia" OR "peripheral artery stenosis" OR "rheumatic heart disease" OR "heart valve diseases" OR "mitral valve stenosis" OR "aortic valve disease" OR "multivalvular disease" OR "valvular heart disease") | 154,762 |
|  | #3 | #1 and #2 with Cochrane Library publication date from Jan 2006 to Jun 2025 | 1,123 |
| CINAHL | #1 | (MM "Digital Health" OR MH "Digital technology" OR MM "Telemedicine“)  OR TX("digital health literacy" OR "eHealth literacy" OR "digital literacy" OR "electronic health literacy" OR "mobile health literacy" OR "digital health" OR "telemedicine" OR "eHealth" OR "digital health technology" OR "medical technology") | 68,204 |
|  | #2 | (MH "Cardiovascular Diseases" OR　MH "Coronary Disease" OR MH "Angina Pectoris" OR　MH "Myocardial Infarction"　OR MH "Heart Failure" OR　MH "Arrhythmia" OR　MH "Atrial Fibrillation" OR　MH "Heart Arrest" OR MH "Rheumatic Heart Disease" OR MH "Heart Valve Diseases") OR　TX("cardiovascular disease" OR "coronary disease" OR "coronary artery disease" OR "angina pectoris" OR "angina" OR "ischemic heart disease" OR "myocardial infarction" OR "heart infarction" OR "heart failure" OR "congestive heart failure" OR "arrhythmias, cardiac" OR "atrial fibrillation" OR "cardiac arrhythmia" OR "heart arrhythmia" OR "arrhythmia" OR "ventricular arrhythmia" OR "heart arrest" OR "cardiac arrest" OR "sudden cardiac death" OR "peripheral arterial disease" OR "intermittent claudication" OR "critical limb ischemia" OR "peripheral artery stenosis" OR "rheumatic heart disease" OR "heart valve diseases" OR "mitral valve stenosis" OR "aortic valve disease" OR "multivalvular disease" OR "valvular heart disease") | 475,342 |
|  | #3 | #1 and #2 with full text, english, peer reviewed, 2006.01.01-2025.06.30 | 4,386 |
| Scopus | #1 | TITLE-ABS-KEY("digital health literacy" OR "eHealth literacy" OR "digital literacy" OR "electronic health literacy" OR "mobile health literacy" OR "digital health" OR "telemedicine" OR "eHealth" OR "digital health technology" OR "medical technology") | 181,708 |
|  | #2 | TITLE-ABS-KEY("cardiovascular disease" OR "coronary disease" OR "coronary artery disease" OR "angina pectoris" OR "angina" OR "ischemic heart disease" OR "myocardial infarction" OR "heart infarction" OR "heart failure" OR "congestive heart failure" OR "arrhythmias, cardiac" OR "atrial fibrillation" OR "cardiac arrhythmia" OR "heart arrhythmia" OR "arrhythmia" OR "ventricular arrhythmia" OR "heart arrest" OR "cardiac arrest" OR "sudden cardiac death" OR "peripheral arterial disease" OR "intermittent claudication" OR "critical limb ischemia" OR "peripheral artery stenosis" OR "rheumatic heart disease" OR "heart valve diseases" OR "mitral valve stenosis" OR "aortic valve disease" OR "multivalvular disease" OR "valvular heart disease") | 2,023,974 |
|  | #3 | #1 and #2 AND PUBYEAR > 2005 AND PUBYEAR < 2026 AND ( LIMIT-TO ( DOCTYPE , "ar" ) ) AND ( LIMIT-TO ( EXACTKEYWORD , "Human" ) OR LIMIT-TO ( EXACTKEYWORD , "Humans" ) OR LIMIT-TO ( EXACTKEYWORD , "Article" ) ) AND ( LIMIT-TO ( LANGUAGE , "English" ) ) AND ( LIMIT-TO ( SRCTYPE , "j" ) ) | 4,033 |
| Web of Science | #1 | TS=("digital health literacy" OR "eHealth literacy" OR "digital literacy" OR "electronic health literacy" OR "mobile health literacy" OR "digital health" OR "telemedicine" OR "eHealth" OR "digital health technology" OR "medical technology") | [81,556](https://www.webofscience.com/wos/woscc/summary/42fe1df2-02da-4602-b1fe-a46db6b2787a-01793831de/relevance/1) |
|  | #2 | TS=("cardiovascular disease" OR "coronary disease" OR "coronary artery disease" OR "angina pectoris" OR "angina" OR "ischemic heart disease" OR "myocardial infarction" OR "heart infarction" OR "heart failure" OR "congestive heart failure" OR "arrhythmias, cardiac" OR "atrial fibrillation" OR "cardiac arrhythmia" OR "heart arrhythmia" OR "arrhythmia" OR "ventricular arrhythmia" OR "heart arrest" OR "cardiac arrest" OR "sudden cardiac death" OR "peripheral arterial disease" OR "intermittent claudication" OR "critical limb ischemia" OR "peripheral artery stenosis" OR "rheumatic heart disease" OR "heart valve diseases" OR "mitral valve stenosis" OR "aortic valve disease" OR "multivalvular disease" OR "valvular heart disease") | [1,278,698](https://www.webofscience.com/wos/woscc/summary/430251d8-037b-40a4-ad2f-269cff56bac2-0179383801/relevance/1) |
|  | #3 | #1 and #2 Refined By:Publication Years: 2014 or 2013 or 2012 or 2011 or 2010 or 2009 or 2008 or 2007 or 2006 or 2015 or 2016 or 2017 or 2018 or 2019 or 2020 or 2021 or 2023 or 2024 or 2025 or 2022. Click to remove this refine from your search.Document Types: Article. Click to remove this refine from your search.Languages: English | 2,357 |
| Google Scholar |  | ehealth literacy and CVD, digital health literacy and CVD | 306 |
