# Supplementary material for: Determinants and Health Outcomes of Digital Health Literacy in Patients With Cardiovascular Disease: Systematic Review and Meta-Analysis
Source: J Med Internet Res. 2026 Mar 24;28:e89102. doi: 10.2196/89102 (PMC13058533; doi:10.2196/89102)
Supplement: Multimedia Appendix 3 [file jmir_v28i1e89102_app3.docx]

# Summary of Digital Health Literacy Measurement Tools Included in This Review

| **Tool** | **Original**  **Authors** | **Year** | **Factor Structure** | **Items** | **Scale** | **Score Range** | **Development Population** | **Reference No. of Included Studies** |
| --- | --- | --- | --- | --- | --- | --- | --- | --- |
| eHEALS (eHealth Literacy Scale) | Norman & Skinner | 2006 | Originally single-factor | 8 items  1. I know what health resources are available on the Internet  2. I know where to find helpful health resources on the Internet  3. I know how to find helpful health resources on the Internet  4. I know how to use the Internet to answer my questions about health  5. I know how to use the health information I find on the Internet to help me  6. I have the skills I need to evaluate the health resources I find on the Internet  7. I can tell high quality health resources from low quality health resources on the Internet  8. I feel confident in using information from the Internet to make health decisions | 5-point Likert  (1 = strongly disagree,  5 = strongly agree) | 8-40 | Adolescents (ages 13-21), Canada | [36], [37],  [38], [42],  [44], [45],  [47], [51],  [53] |
| Researcher-developed Digital Resources Questionnaire | Rodríguez Parrado et al | 2022 |  | 9 items  1. Is there any support you can rely on from a relative or caregiver when using technological resources?  2. Do you feel comfortable when asking your caregiver or relative for help with the use of technological resources?  3. Do you have sufficient knowledge and skills for the use of Internet, chat, forums, and health applications?  4. How often do you try to resolve doubts about your illness using the Internet?  5. Do your family and friends encourage you to use the Internet, applications, forums, and blogs to improve your self-care?  6. Are you motivated to receive training to improve self-care through the Internet, chat, apps, forums, and blogs?  7. Do you feel safe and confident when accessing to the Internet, chats, applications, forums, and health blogs?  Technological resource  (Smartphone / Social networks / Classic, basic cell phone / Landline)  Time per day  (Does not spend time / 1 h / Between 2 and 3 h / More than 3 h) | 4-point Likert  (1 = always,  4 = never) | 7-28^a^ | Heart failure patients  (mean age 67.3 ± 12.9 years), Colombia | [39] |
| eHLQ (eHealth Literacy Questionnaire) | Kayser et al | 2018 | 7-Factor | 7-Factor (35 items)  Factor 1. Using technology to process health information  Factor 2. Understanding of health concepts and language  Factor 3. Ability to actively engage with digital services  Factor 4. Feel safe and in control  Factor 5. Motivated to engage with digital services  Factor 6. Access to digital services that work  Factor 7. Digital services that suit individual needs | 4-point Likert (1 = strongly disagree, 4 = strongly agree) | 35-140 | Adults  (≥18 years), Denmark | [40] |
| Researcher-developed ICT^b^ literacy item | Yun et al | 2022 |  | Do you have any difficulties in using new technologies | 5-point Likert (1 = no difficulty at all, 5 = maximum difficulty) | 1-5 (later dichotomized: 1-2 = higher ICT skills, 3-5 = lower ICT skills) | Heart failure patients (Median: 74), Spain | [41] |
| Internet anxiety, digital confidence, prior experiences with mHealth interventions | NR^c^ | NR |  | **Internet anxiety (3 items)**  I have concerns about using the internet  (remaining items not reported)  **Digital confidence (3 items)**  Items not reported  **Prior experiences with mHealth interventions (1 item)**  Item not reported | **Internet anxiety:**  5-point Likert (1 = does not apply to me, 5 = does apply to me)  **Digital confidence:** 5-point Likert (1 = not confident at all, 5 = very confident) | **Internet anxiety∙ Digital confidence:**  3-15 | NR | [43] |
| Composite tool | Rahman et al | 2016 | 4-Factor | 4-Factor (30 items)  Factor 1. General self-efficacy  Factor 2. Computer self-efficacy  Factor 3. Health technology self-efficacy  Factor 4. Attitude toward health technology | 7-point Likert (1 = strongly disagree, 5 = strongly agree) | 30-150 | Undergraduate and graduate students, United States | [46] |
| DHLI (Digital Health Literacy Instrument) | van der Vaart et al | 2017 | 4-Factor | Self-report items (7-Factor, 21 items)  Factor 1. Operational skills  Factor 2. Navigation skills  Factor 3. Information searching  Factor 4. Evaluating reliability  Factor 5. Determining relevance  Factor 6. Adding self-generated content  Factor 7. Protecting privacy  Performance-based items (7 items)^d^  1. If you were to minimalize this page, to open another program on your computer. Which button would you use?  2. Then, you want to leave this website and go back to your search results in Google. Which button would you use?  3. What kind of information do you expect to find when you click on button A?  4. You want to know who are behind the foundation. Which button would you click on?  5. Which of these results would most likely give you a correct and reliable answer?  6. Which ofthe messages takes privacy into account properly  7. Write down below what you would ask your doctor in this situation | 4-point Likert (1 = very easy or never, 4 = very difficult or often)  5-item multiple-choice response format | 21-84  0-7 | Adults  (≥18 years), Netherlands | [48], [50] |
| Internet anxiety, digital confidence, digital overload | NR | NR |  | **Internet anxiety (3 items)**  I have concerns about using the internet  **Digital confidence (3 items)**  How confident are you in using digital media?  **Digital overload (3 items)**  I feel burdened by the constant accessibility via cell phone or e-mail  (remaining items not reported) | **Internet anxiety∙ Digital overload:**  5-point Likert (1 = strongly disagree, 5 = strongly agree)  **digital confidence:** 5-point Likert (1 = not very confident, 5 = very confident) | 3–15 (each scale) | NR | [49] |
| DHRQ (Digital Health Readiness Questionnaire) | Scherrenberg et al | 2023 | 4-Factor, 1-additional category | **4-Factor (15 items)**  Factor 1. Digital access  Factor 2. Usage of digital technology  Factor 3. Digital literacy  Factor 4. Digital health literacy  **1-additional category (5 items)^e^**  Learnability: Motivation and interest to engage with new technology. | 5-point Likert (1 = strongly disagree, 5 = strongly agree) | 15-75 | Cardiology outpatients (≥18 years), Belgium | [52] |

^a^Items 1–7: Likert scale (range 7–28), total score based on sum.

^b^ICT: information and communications technology.

^c^NR: not reported.

^d^Example items are abridged versions of the original performance-based tasks.

^e^Digital learnability is assessed separately (5–25).
