# Supplementary material for: Determinants and Health Outcomes of Digital Health Literacy in Patients With Cardiovascular Disease: Systematic Review and Meta-Analysis
Source: J Med Internet Res. 2026 Mar 24;28:e89102. doi: 10.2196/89102 (PMC13058533; doi:10.2196/89102)
Supplement: Multimedia Appendix 4 [file jmir_v28i1e89102_app4.docx]

**Quality Assessment for Cross-Sectional Studies Using the AXIS Tool**

| Study | AXIS^a^ quality appraisal items (1–20) | | | | | | | | | | | | | | | | | | | | Total |
| --- | --- | --- | --- | --- | --- | --- | --- | --- | --- | --- | --- | --- | --- | --- | --- | --- | --- | --- | --- | --- | --- |
|  | 1 | 2 | 3 | 4 | 5 | 6 | 7 | 8 | 9 | 10 | 11 | 12 | 13 | 14 | 15 | 16 | 17 | 18 | 19 | 20 |  |
| Chuang et al (2019) [46] | Yes | Yes | No | Yes | Yes | Yes | Yes | Yes | Yes | Yes | Yes | Yes | No | Yes | Yes | Yes | Yes | Yes | Yes | Yes | 18 |
| Bakhshayesh et al (2023) [51] | Yes | Yes | Yes | Yes | Yes | Yes | DNK^b^ | Yes | Yes | Yes | Yes | Yes | No | DNK | Yes | Yes | Yes | Yes | Yes | Yes | 17 |
| Bäuerle et al (2023) [52] | Yes | Yes | No | Yes | Yes | Yes | DNK | Yes | Yes | Yes | Yes | Yes | Yes | No | Yes | Yes | Yes | Yes | Yes | Yes | 17 |
| Rush et al (2023) [55] | Yes | Yes | No | Yes | Yes | Yes | No | Yes | Yes | Yes | Yes | Yes | Yes | Yes | Yes | Yes | Yes | Yes | Yes | Yes | 18 |
| Vitolo et al (2023) [57] | Yes | Yes | No | Yes | Yes | Yes | No | Yes | Yes | Yes | Yes | Yes | DNK | No | Yes | Yes | Yes | Yes | Yes | Yes | 16 |
| Mohajeri et al (2024) [58] | Yes | Yes | No | Yes | Yes | Yes | Yes | Yes | Yes | Yes | Yes | Yes | No | Yes | Yes | Yes | Yes | Yes | Yes | Yes | 18 |
| Astuti et al (2025) [60] | Yes | Yes | Yes | Yes | Yes | Yes | DNK | Yes | Yes | Yes | Yes | Yes | No | DNK | Yes | Yes | Yes | Yes | Yes | Yes | 17 |
| Cuppen et al (2025) [61] | Yes | Yes | DNK | Yes | Yes | Yes | DNK | Yes | Yes | Yes | Yes | Yes | No | DNK | Yes | Yes | Yes | Yes | Yes | Yes | 16 |
| Zhao et al (2025) [62] | Yes | Yes | DNK | Yes | Yes | Yes | Yes | Yes | Yes | Yes | Yes | Yes | No | Yes | Yes | Yes | Yes | Yes | Yes | Yes | 18 |
| Dibek et al (2025) [63] | Yes | Yes | Yes | Yes | Yes | Yes | No | Yes | Yes | Yes | Yes | Yes | No | No | Yes | Yes | Yes | Yes | Yes | Yes | 17 |
| Sun et al (2025) [64] | Yes | Yes | Yes | Yes | Yes | Yes | No | Yes | Yes | Yes | Yes | Yes | No | No | Yes | Yes | Yes | Yes | Yes | Yes | 17 |

^a^AXIS = appraisal tool for cross-sectional studies.

^b^DNK: do not know.
