# Supplementary material for: Determinants and Health Outcomes of Digital Health Literacy in Patients With Cardiovascular Disease: Systematic Review and Meta-Analysis
Source: J Med Internet Res. 2026 Mar 24;28:e89102. doi: 10.2196/89102 (PMC13058533; doi:10.2196/89102)
Supplement: Multimedia Appendix 5 [file jmir_v28i1e89102_app5.docx]

**Newcastle–Ottawa Quality Assessment of Longitudinal and Prospective Observational Studies**

| Criteria | Selection | | | | Comparability | Outcome | | | Total |
| --- | --- | --- | --- | --- | --- | --- | --- | --- | --- |
|  | Representativeness of the exposed cohort | Selection of the non-exposed cohort | Ascertainment of exposure | Demonstration that outcome of interest was not present at start of study | Comparability of cohorts on the basis of the design or analysis controlled for confounders | Assessment of outcome | Was follow-up long enough for outcomes to occur | Adequacy of follow-up of cohorts |  |
| Lin et al (2020) [47] | ★ |  | ★ | ★ | ★ | ★ | ★ | ★ | 7 |
| Brørs et al (2023) [53] | ★ |  | ★ | ★ | ★ | ★ | ★ |  | 6 |
| Ramstad et al (2023) [54] | ★ |  | ★ | ★ | ★ |  | ★ |  | 5 |
| van Schalkwijk et al (2024) [59] | ★ |  | ★ | ★ | ★ |  | ★ |  | 5 |
