# Supplementary material for: Determinants and Health Outcomes of Digital Health Literacy in Patients With Cardiovascular Disease: Systematic Review and Meta-Analysis
Source: J Med Internet Res. 2026 Mar 24;28:e89102. doi: 10.2196/89102 (PMC13058533; doi:10.2196/89102)
Supplement: Multimedia Appendix 6 [file jmir_v28i1e89102_app6.docx]

**Risk-of-Bias Tool for Randomized Controlled Trials**

| Study | RoB 2 quality appraisal items (domain 1–5) | | | | | | | | | | | | | | | | | | | | | |  |
| --- | --- | --- | --- | --- | --- | --- | --- | --- | --- | --- | --- | --- | --- | --- | --- | --- | --- | --- | --- | --- | --- | --- | --- |
|  | Domain 1 | | | Domain 2 | | | | | | | Domain 3 | | | | Domain 4 | | | | | Domain 5 | | | Overall |
|  | 1 | 2 | 3 | 1 | 2 | 3 | 4 | 5 | 6 | 7 | 1 | 2 | 3 | 4 | 1 | 2 | 3 | 4 | 5 | 1 | 2 | 3 |  |
| Spindler et al (2022) [49] | Y^a^ | NI | N | NI | NI | N | NA | NA | Y | N | N | N | N | NA | N | N | NI | PN | NA | Y | N | N | High |
| Yun et al (2022) [50] | Y | NI | NI | NI | NI | NI | NA | NA | Y | NA | Y | NA | NA | NA | N | N | NI | N | NA | NI | Y | Y | Some concerns |

^a^Legend: Y = Yes; PY = Probably yes; PN = Probably no; N = No; NA = Not applicable; NI = No information.
