# Supplementary material for: Determinants and Health Outcomes of Digital Health Literacy in Patients With Cardiovascular Disease: Systematic Review and Meta-Analysis
Source: J Med Internet Res. 2026 Mar 24;28:e89102. doi: 10.2196/89102 (PMC13058533; doi:10.2196/89102)
Supplement: Multimedia Appendix 7 [file jmir_v28i1e89102_app7.docx]

**Risk of Bias in Nonrandomized Studies of Interventions**

| Study | ROBINS-I quality appraisal items (domain 1–7) | | | | | | | | | | | | | | | | | | | | | | | | | | | | | | | | | | | | | |  | |
| --- | --- | --- | --- | --- | --- | --- | --- | --- | --- | --- | --- | --- | --- | --- | --- | --- | --- | --- | --- | --- | --- | --- | --- | --- | --- | --- | --- | --- | --- | --- | --- | --- | --- | --- | --- | --- | --- | --- | --- | --- |
|  | Domain 1 | | | | | | | | Domain 2 | | | | | Domain 3 | | | Domain 4 | | | | | | | Domain 5 | | | | | | Domain 6 | | | | | Domain 7 | | | | Judgement | |
|  | 1 | 2 | 3 | 4 | 5 | 6 | 7 | 8 | 1 | 2 | 3 | 4 | 5 | 1 | 2 | 3 | 1 | 2 | 3 | 4 | 5 | 6 | 1 | | 2 | 3 | 4 | 5 | 1 | | 2 | 3 | 4 | 1 | | 2 | 3 |  | |  |
| Melholt et al (2018) [45] | PY^a^ | NA | N | Y | Y | PN | NA | NA | N | NA | NA | Y | NA | Y | Y | N | N | NA | Y | Y | Y | NA | Y | | N | PN | PY | PY | N | | PY | Y | N | PN | | PN | PN | Moderate | |  |
| Rodríguez Parrado et al (2022) [48] | PY | NA | NA | PY | PY | NA | NA | NA | N | NA | NA | Y | NA | Y | Y | N | N | NA | Y | Y | PY | NA | PY | | NA | PN | PY | PY | PN | | PY | Y | N | PN | | PN | PN | Moderate | |  |
| Son et al (2023) [56] | PY | NA | NA | PY | PY | PN | NA | NA | N | NA | NA | Y | NA | Y | Y | N | PN | NA | PY | Y | PY | NA | PY | | N | PN | PY | PY | PN | | PY | Y | N | PN | | PN | PN | Moderate | |  |

^a^Legend: Y = Yes; PY = Probably yes; PN = Probably no; N = No; NA = Not applicable; NI = No information.
