# Supplementary material for: Determinants and Health Outcomes of Digital Health Literacy in Patients With Cardiovascular Disease: Systematic Review and Meta-Analysis
Source: J Med Internet Res. 2026 Mar 24;28:e89102. doi: 10.2196/89102 (PMC13058533; doi:10.2196/89102)
Supplement: Multimedia Appendix 8 [file jmir_v28i1e89102_app8.docx]

# Study-Level Digital Health Literacy Levels, Associated Factors, and Health Outcomes

| **Authors (year)** | **DHL^a^**  **measurement** | **DHL level**  **(total or item means)** | **Associated factors** | **Health outcome** |
| --- | --- | --- | --- | --- |
| Melholt et al (2018) [45] | eHEALS^b^ | 28.80±5.96^c^ (Baseline)  30.88±5.17^c^ (After 3 months) | Higher education level was associated with higher eHEALS  (eHEALS items, *p* = .005–0.023).  More frequent computer use was associated with higher e-Health literacy  (eHEALS items, *p* = .004–0.046).  More frequent Internet use was associated with higher e-Health literacy  (eHEALS items, *p* < .001–0.043). |  |
| Chuang et al (2019) [46] | eHEALS | 26.2±5.7 |  | Positively correlated with social support (r = .24, *p* < .01).  Positively correlated with HF knowledge (r = .50, *p* < .01).  Positively correlated with self-care management (r = .25, *p* < .01).  Directly and indirectly associated with self-care management (β = .17, *p* < .05; β = .04, *p* < .05)  Indirectly associated with self-care maintenance (β = .04, *p* < .05) |
| Lin et al  (2020) [47] | eHEALS | 28.16±5.46 |  | Medication adherence (MARS-5^d^): B = 0.63, *p* < .001  Quality of life (MLHFQ^e^): B = –0.49, *p* < .001  Cardiac events: HR = 0.31, *p* = .013 (69% risk reduction) |
| Rodríguez Parrado et al  (2022) [48] | Researcher-developed questionnaire | 2.33±1.25 (Baseline)^f^  1.93±0.82 (After 8 sessions)^f^ |  | DHL (knowledge and skills for digital resources, resolving doubts via the Internet, and family/friend motivation): Improved after the DHL program (*p* = .003; *p* = .001; *p* = .001).  Empowerment (overall score): Significantly improved from 27.6 to 38.2 (*p* = .001) after the DHL program. |
| Spindler et al  (2022) [49] | eHLQ^g^ | Intervention group: item means 2.98-3.22  Control group: item means 2.71-3.17  (After 6 months)^h^  Intervention group: item means 3.01-3.27  Control group: item means 2.78-3.22  (After 12 months) |  | Improved technology use & digital service motivation after 6 months (d = 0.42, *p* = .04; d = 0.59, p < .001) |
| Yun et al (2022) [50] | Researcher-developed questionnaire | 72% lower ICT^i^ skills (n=128);  28% higher ICT skills (n=50) | **Demographic factors**  Older age, female sex, and lower education (age: 76 ± 10 vs. 69 ± 12 years, *p* < .001; females: 47% vs. 26%, *p* = .012; middle school or higher education: 22% vs. 48%, *p* = .002; low vs. middle/higher ICT skills group).  **Clinical factors**  Lower BMI^j^, lower DBP^k^, lower Hb^l^ ,higher NT-proBNP^m^ levels, and higher NYHA^n^ functional class (BMI: 28 ± 5 vs. 29 ± 5, *p* = .047; DBP: 68 ± 13 vs. 72 ± 15 mmHg, *p* = .043; Hb: 12 ± 3 vs. 13 ± 2 g/dl, *p* = .003; NT-proBNP: 1672 pg/ml vs. 1348 pg/ml, *p* = .011; NYHA class III–IV: 56% vs. 22%, *p* < .001; low vs. middle/higher ICT skills group).  **Functional and cognitive factors**  Greater functional dependence, Lower cognitive function (Lawton and Brody scale: 14 ± 6 vs. 11 ± 4, *p* = .003; MMSE^o^: 25 ± 4 vs. 27 ± 4, *p* = .004; low vs. middle/higher ICT skills group).  **Psychosocial factors**  Higher social support, Greater need a caregiver (Duke-UNC Functional Social Support Questionnaire: 52 ± 7 vs. 47 ± 11, *p* = .01; needs a caregiver: 31% vs. 10%, *p* = .004; low vs. middle/higher ICT skills group). |  |
| Bakhshayesh et al (2023) [51] | eHEALS | 18.09±9.08 | Older age was associated with lower eHEALS  (age >50 years: eHEALS mean 16.36 vs. age ≤50 years: 24.06, *p* < .001).  Lower education level was associated with lower eHEALS  (below diploma: eHEALS mean 8.57 vs. master’s degree or higher: 31.26, *p* < .001). | Positively correlated with self-care behaviors (r = .54, *p* = .001)  Positively correlated with quality of life (r = .50, *p* = .001)  Positively associated with quality of life (β = .16, *p* = .043) |
| Bäuerle et al (2023) [52] | Internet anxiety, Digital confidence, Prior experiences with mHealth interventions | NR |  | Digital confidence positively predicted acceptance (β = 0.19, *p* = .003). |
| Brørs et al (2023) [53] | eHEALS | 27.27±6.28 (Baseline)  26.97±5.98 (After 12 months) |  | **[Baseline]** Higher eHEALS skills scores were associated with fewer depressive symptoms (β = –0.88, *p* = .005).  Higher eHEALS skills scores were associated with more physical activity (β = 0.11, *p* = .007).  Higher eHEALS evaluate scores were associated with lower odds of current smoking (OR = 0.92, *p* = .030).  **[12 months]** Higher eHEALS evaluate scores were associated with fewer depressive symptoms (β = –0.64, *p* = .003) and fewer anxiety symptoms (β = –0.75, *p* = .001). |
| Ramstad et al (2023) [54] | eHEALS | 25.71±6.22 (Baseline) | Use of the internet and the national health portal was associated with higher eHealth literacy  (internet use to find health information: coefficient 10.90, *p* = .001; national health portal use: coefficient 3.79, *p* = .029; portal use to find health information: coefficient 5.42, *p* = .001; portal use to find information about patient rights/own health information: coefficient 3.06, *p* = .004). | **[2 months]** Higher eHEALS was associated with greater internet use (OR = 1.19, *p* < .001) and health app use (OR = 1.15, *p* < .001). |
| Rush et al (2023) [55] | Composite tool (General self-efficacy, Computer self-efficacy, Health technology self-efficacy, Attitude toward health technology) | 5.38±1.27  (Computer self-efficacy)  5.63±1.01  (Health technology self-efficacy)  5.46±0.87  (Attitude toward health technology) | Female was associated with lower self-efficacy and health care technology attitudes scale  (computer self-efficacy: 5.63 vs. 4.95, p < .001; health technology self-efficacy: 5.81 vs. 5.32, *p* < .001; technology attitude: 5.66 vs. 5.13, *p* < .001). | Higher health technology self-efficacy was associated with more positive attitudes toward health care technology (β=0.62, p<.001).  More positive attitudes toward health care technology were associated with higher telehealth satisfaction (β = 0.47, *p* < .001).  Higher computer self-efficacy was associated with lower telehealth satisfaction (β = –0.16, p = .01) |
| Son et al (2023) [56] | eHEALS | Intervention group: 24.32±9.55  Control group: 25.58±9.79 (Baseline)  Intervention group: 26.14±9.74  Control group: 24.12±8.72 (After 1 month)  Intervention group: 26.24±9.76  Control group: 25.48±8.53 (After 3 months)  Intervention group: 28.10±9.16  Control group: 27.20±8.73 (After 6 months) | Older age was associated with lower eHEALS  (age <60 years: eHEALS mean 27.74 vs. age ≥60 years: 21.41, *p* = .015) |  |
| Vitolo et al (2023) [57] | DHLI^p^ | 48.58±24.16 | Higher frailty status was associated with lower DHL (higher DHLI total scores)  (DHLI total score: robust 44.86 ± 22.76 vs. pre-frail 53.94 ± 25.53 vs. frail 61.66 ± 24.51, *p* < .001). |  |
| Mohajeri et al (2024) [58] | Internet anxiety, Digital confidence, Digital overload | 1.58±0.80 (Internet anxiety)  3.87±0.98 (Digital confidence)  1.94±1.01 (Digital overload) |  | Internet anxiety was negatively associated with mHealth acceptance (β = –0.20, *p* = .004), while digital confidence was positively associated (β = 0.25, *p* < .001). |
| Van Schalkwijk et al (2024) [59] | DHLI | Item mean 2.97±0.60 |  | Higher DHL associated with greater application of e-health (OR = 2.22, *p* < .001) |
| Astuti et al (2025) [60] | eHEALS | 26.35±7.40 |  | eHEALS was positively associated with self-care (B = 0.31, *p* = .002) |
| Cuppen et al (2025) [61] | DHRQ^q^ | 38.4 ± 17.7 |  | DHRQ was positively associated with willingness to participate in telemonitoring (B = 0.089, p = .01) |
| Zhao et al (2025) [62] | eHEALS | 18.18 ± 10.11 | Lower social support was associated with lower eHEALS (β = 0.445, *p* < .001).  Rural residence was associated with lower eHEALS (β = –0.099, *p* < .05).  Lower education level was associated with lower eHEALS (β = 0.290, *p* < .001). | eHEALS was positively associated with healthcare technology self-efficacy (β = 0.661, *p* < .001).  eHEALS was negatively associated with technophobia (β = –0.152, *p* < .05). |

^a^DHL: digital health literacy.

^b^eHEALS:ehealth literacy scale.

^c^Scores calculated by authors.

^d^MARS-5: 5-item medication adherence report scale.

^e^Lower scores on the Minnesota Living with Heart Failure Questionnaire (MLHFQ) indicate better quality of life.

^f^Scores calculated by authors; Lower scores indicate greater knowledge about digital resources applied to health.

^g^eHLQ: ehealth literacy questionnaire.

^h^Baseline data were not reported in this study.

^i^ICT: information and communications technology.

^j^BMI: body mass index.

^k^DBP: diastolic blood pressure.

^l^Hb: hemoglobin.

^m^NT-proBNP: N-terminal pro-B-type natriuretic peptide.

^n^NYHA: new york heart association classification of heart failure functional status.

^o^MMSE: mini-mental state examination.

^p^DHLI: digital health literacy instrument.

^q^DHRQ: digital health-related questionnaire.
