# Supplementary material for: Determinants and Health Outcomes of Digital Health Literacy in Patients With Cardiovascular Disease: Systematic Review and Meta-Analysis
Source: J Med Internet Res. 2026 Mar 24;28:e89102. doi: 10.2196/89102 (PMC13058533; doi:10.2196/89102)
Supplement: Multimedia Appendix 9 [file jmir_v28i1e89102_app9.docx]

**GRADE Assessment**

| Outcomes | No. of studies | Study design | Risk of Bias | Inconsistency | Indirectness | Imprecision | Publication bias | Certainty |
| --- | --- | --- | --- | --- | --- | --- | --- | --- |
| Disease knowledge | 1 | Cross-sectional | Not serious | Not serious^a^ | Not serious | Serious^b^ | Not serious | ⨁◯◯◯ |
| Empowerment | 1 | Quasi-experimental | Not serious^c^ | Not serious^a^ | Not serious | Serious^b^ | Not serious | ⨁◯◯◯ |
| Smoking | 1 | Prospective | Not serious | Not serious^a^ | Not serious | Serious^b^ | Not serious | ⨁◯◯◯ |
| Self-care behaviors | 3 | Cross-sectional | Not serious | Not serious | Not serious | Serious^b^ | Not serious | ⨁◯◯◯ |
| Physical activity | 1 | Prospective | Not serious | Not serious^a^ | Not serious | Serious^b^ | Not serious | ⨁◯◯◯ |
| Medication adherence | 1 | Longitudinal | Not serious | Not serious^a^ | Not serious | Serious^b^ | Not serious | ⨁◯◯◯ |
| Cardiac events | 1 | Longitudinal | Not serious | Not serious^a^ | Not serious | Serious^b^ | Not serious | ⨁◯◯◯ |
| Symptom status | 1 | Cross-sectional | Not serious | Not serious^a^ | Not serious | Serious^b^ | Not serious | ⨁◯◯◯ |
| Depression | 1 | Prospective | Not serious | Not serious^a^ | Not serious | Serious^b^ | Not serious | ⨁◯◯◯ |
| Anxiety | 1 | Prospective | Not serious | Not serious | Not serious | Serious^b^ | Not serious | ⨁◯◯◯ |
| Quality of life | 1 | Longitudinal | Not serious | Not serious^a^ | Not serious | Serious^b^ | Not serious | ⨁◯◯◯ |
|  | 1 | Cross-sectional | Not serious | Not serious^a^ | Not serious | Serious^b^ | Not serious | ⨁◯◯◯ |
| Use of digital health technology | 1 | Randomized controlled trial | Serious^d^ | Not serious^a^ | Not serious | Serious^b^ | Not serious | ⨁⨁◯◯ |
|  | 1 | Prospective,  Longitudinal | Serious^e^ | Not serious | Not serious | Serious^b^ | Not serious | ⨁◯◯◯ |
|  | 1 | Cross-sectional | Not serious | Not serious^a^ | Not serious | Serious^b^ | Not serious | ⨁◯◯◯ |
| Acceptance of digital health | 2 | Cross-sectional | Not serious | Not serious | Not serious | Serious^b^ | Not serious^f^ | ⨁◯◯◯ |
|  | 1 | Cross-sectional | Not serious | Not serious^a^ | Not serious | Serious^b^ | Not serious | ⨁◯◯◯ |
|  | 1 | Cross-sectional | Not serious | Not serious^a^ | Not serious | Serious^b^ | Not serious | ⨁◯◯◯ |

^a^ Only one study was included.

**^b^** The number of studies was limited (1–3 studies), sample sizes were small, and no pooled estimate was available, resulting in substantial uncertainty regarding the precision of the effect.
**^c^** Outcome assessors may have been aware of participants’ exposure or intervention status; however, this was unlikely to have meaningfully biased the results.
**^d^** The randomization process and blinding procedures were not clearly reported, indicating a potential risk of performance and detection bias.
**^e^** Outcomes were primarily self-reported, and reporting on follow-up rates or attrition was insufficient.

**^f^** Both statistically significant results and null effects were reported, suggesting a low likelihood of selective publication.
